# Genetic Predisposition to Breast and Ovarian Cancers: How Many and Which Genes to Test?

**DOI:** 10.3390/ijms21031128

**Published:** 2020-02-08

**Authors:** Davide Angeli, Samanta Salvi, Gianluca Tedaldi

**Affiliations:** 1 Biostatistics and Clinical Trials Unit, Istituto Scientifico Romagnolo per lo Studio e la Cura dei Tumori (IRST) IRCCS, 47014 Meldola, Italy; davide.angeli@irst.emr.it; 2 Biosciences Laboratory, Istituto Scientifico Romagnolo per lo Studio e la Cura dei Tumori (IRST) IRCCS, 47014 Meldola, Italy; samantasalvi@gmail.com

**Keywords:** hereditary breast and ovarian cancer, gene panels, cancer predisposition, next-generation sequencing, cancer risk

## Abstract

Breast and ovarian cancers are some of the most common tumors in females, and the genetic predisposition is emerging as one of the key risk factors in the development of these two malignancies. *BRCA1* and *BRCA2* are the best-known genes associated with hereditary breast and ovarian cancer. However, recent advances in molecular techniques, Next-Generation Sequencing in particular, have led to the identification of many new genes involved in the predisposition to breast and/or ovarian cancer, with different penetrance estimates. *TP53*, *PTEN*, *STK11*, and *CDH1* have been identified as high penetrance genes for the risk of breast/ovarian cancers. Besides them, *PALB2*, *BRIP1*, *ATM*, *CHEK2*, *BARD1*, *NBN*, *NF1*, *RAD51C*, *RAD51D* and mismatch repair genes have been recognized as moderate and low penetrance genes, along with other genes encoding proteins involved in the same pathways, possibly associated with breast/ovarian cancer risk. In this review, we summarize the past and more recent findings in the field of cancer predisposition genes, with insights into the role of the encoded proteins and the associated genetic disorders. Furthermore, we discuss the possible clinical utility of genetic testing in terms of prevention protocols and therapeutic approaches.

## 1. Introduction

Breast cancer (BC) and ovarian cancer (OC) are the first and the eighth most common tumors for both incidence and mortality in females, respectively [1]. BC can also affect males, even if male breast cancer (MBC) represents less than 1% of all BCs [2,3,4].

As for other cancers, BC and OC are the result of a combination of both environmental and genetic risk factors. About 10–30% of BCs and OCs show familial clustering, but only 5–10% of cases are estimated to be hereditary, being associated with a germline pathogenic variant (PV) or likely-pathogenic variant (LPV) in a cancer predisposition gene [5,6].

*BRCA1* [7] and *BRCA2* [8] are the main genes involved in Hereditary Breast and Ovarian Cancer syndrome (HBOC) [9], but other genes have also been associated with BC and OC risk [10,11,12,13,14,15,16,17].

In the last few years, the advent of Next-Generation Sequencing (NGS) has enabled the analysis of a great number of genes with the advantage of lower costs and wider access to molecular tests for patients with suspected genetic syndromes [18].

In this complex scenario, one of the main issues is to define how many and which genes should be tested in patients with a suspicion of a genetic predisposition to cancer.

In this review, we summarize the past and most recent genetic findings on BC/OC predisposition, grouping the genes on the basis of their penetrance, calculated on large case-control studies, and taking into account their function and association with genetic disorders (Table 1). The penetrance of a disease-causing genetic variant is the proportion of carriers of that variant who develop the disease, whereas the relative risk (RR) is the measure of the risk of developing a disease compared to the risk of the general population. A genetic variant is usually defined with high penetrance when the RR for the carrier is ≥10.0, with medium-high penetrance when the RR is between 5.0 and 10.0, with moderate penetrance when the RR is between 2.0 and 5.0, and with low penetrance when the RR is between 1.0 and 2.0 [19].

## 2. High Penetrance Genes

### 2.1. BRCA1 and BRCA2

The *BRCA1* gene is located on chromosome 17q21.31 and encodes a nuclear protein involved in DNA repair, cell cycle checkpoint control, and maintenance of genomic stability [39,40]. The BRCA1 protein is a tumor suppressor acting with other tumor suppressors, DNA damage sensors, and signal transducers to form a large multi-subunit protein complex known as BRCA1-associated genome surveillance complex (BASC) [41,42].

Germline PVs in *BRCA1* gene are associated with a 57–65% and 39–44% risk of developing BC and OC by the age of 70, respectively [20,21,22]. *BRCA1* PV/LPVs have also been associated with an increased risk of BC in males, which is estimated to be 1.2% by the age of 70 [43]. In addition, *BRCA1* PV/LPVs have been associated with an increased risk of colon cancer [44], prostate cancer [45], and pancreatic cancer [46,47].

The *BRCA2* gene is located on chromosome 13q13.1 and encodes a nuclear protein involved in repairing damaged DNA through homologous recombination (HR) [39,40]. BRCA2 protein mediates the recruitment of the recombinase RAD51 to the DNA double-strand breaks (DSBs) through the formation of a BRCA1-PALB2-BRCA2 complex. The BRCA2 protein contains a helical domain, three oligonucleotide binding domains, and a tower domain, which allow BRCA2 binding to both single-stranded DNA and double-stranded DNA [39,48,49].

Germline PV/LPVs in the *BRCA2* gene are associated with a 45–55% and 11–18% risk of developing BC and OC by the age of 70, respectively [20,21,22]. *BRCA2* PV/LPVs have also been associated with an increased risk of BC in males, which is estimated at 6.8% by the age of 70 [43]. In addition, *BRCA2* PV/LPVs have been associated with an increased risk of prostate cancer [50], pancreatic cancer [47,51], and uveal melanoma [52,53].

According to the National Comprehensive Cancer Network (NCCN) guidelines, women with *BRCA1/2* PV/LPVs should undergo a surveillance protocol, including clinical breast examination every 6–12 months and annual breast magnetic resonance imaging (MRI), starting at the age of 25, annual mammography with consideration of tomosynthesis, starting at the age of 30, and annual transvaginal ultrasound and serum CA-125 concentration, although of uncertain benefit, beginning at age 30–35 years [54]. Moreover, they should evaluate the opportunity of a bilateral risk-reducing mastectomy (RRM) and of a bilateral risk-reducing salpingo-oophorectomy (RRSO), typically at between 35 and 40 years and upon completion of childbearing [54]. Men with *BRCA1/2* PV/LPVs should undergo clinical breast examination every 6–12 months, starting at the age of 35, and annual prostate cancer screening, starting at the age of 40 (in particular in *BRCA2* PV/LPV carriers) [55]. In both sexes, screening for melanoma and pancreatic cancer should be evaluated on the basis of family history [54]. Regarding the therapeutic approach, patients with germline *BRCA1/2* variants can benefit from treatment with inhibitors of the poly adenosine-diphosphate ribose polymerase (PARP), that acts through the mechanism of synthetic lethality [56]. Moreover, somatic *BRCA1/2* alterations are present in many sporadic cancers, in particular BCs and OCs, and even these tumors can be treated with PARP inhibitors, having the same molecular characteristics of hereditary cancers [57].

### 2.2. TP53

The *TP53* gene is located on chromosome 17p13.1 and encodes the p53 protein, a tumor suppressor that responds to different cellular stresses to regulate expression of target genes, thereby inducing cell cycle arrest, apoptosis, senescence, DNA repair, or metabolism changes [58]. Due to its crucial function in maintaining the genomic stability, p53 has been defined ”the guardian of the genome” and, indeed, somatic alterations of *TP53* gene are present in about 50% of sporadic tumors [59], conferring to p53 an important role as a biomarker for the diagnosis, tumor progression, poor prognosis, and reduced sensitivity for anticancer drugs [60]. Moreover, p53 is a potential target for therapies because the restoration of its activity has shown to lead to tumor regression [61].

Germline PV/LPVs in the *TP53* gene are associated with Li-Fraumeni syndrome (LFS), a rare autosomal dominant disorder characterized by a high predisposition to several types of cancer, such as brain tumors, sarcomas, acute leukemia, and adrenocortical tumors [25,62,63,64,65,66,67,68,69]. The lifetime risk of BC for patients with LFS is estimated to be 25–79% [70,71,72]. Patients with LFS also have an increased risk of developing genitourinary cancers, including OC, but this risk has not been estimated precisely [17,73]. Given the high risk of developing BC, the NCCN guidelines suggest that women with *TP53* PV/LPVs should undergo clinical breast examination every 6–12 months starting at the age of 20, annual breast MRI screening with contrast from 20 to 75 years, and mammography with consideration of tomosynthesis from 30 to 75 years. After 75 years, the BC risk management should be considered on an individual basis [54]. Moreover, for women with a germline *TP53* PV/LPV, RRM is an option that should be discussed [54].

### 2.3. PTEN

The *PTEN* gene is located on chromosome 10q23.31 and encodes a phosphatidylinositol-3,4,5-trisphosphate 3-phosphatase, which antagonizes the PI3K signaling pathway through its lipid phosphatase activity and negatively regulates the MAPK (mitogen-activated protein kinase) pathway through its protein phosphatase activity [74]. PTEN is one of the most frequently disrupted tumor suppressors in cancer and, for this reason, therapeutic strategies aimed at tumors with loss of PTEN function are under evaluation [75].

Germline PV/LPVs in the *PTEN* gene are associated with the Cowden syndrome (CS), an autosomal dominant disorder characterized by benign hamartomas as well as by an increased lifetime risk of breast, thyroid, uterine, and other cancers [24,76]. The lifetime risk of BC in *PTEN* PV/LPV carriers is estimated to be 25–85%, while the risk of OC is low or none [77,78,79,80,81,82,83]. In accordance with the NCCN guidelines, women with *PTEN* PV/LPVs should undergo clinical breast examination every 6–12 months starting at the age of 25 (or 5–10 years before the earliest known BC in the family), annual mammography with consideration of tomosynthesis and breast MRI screening with contrast starting at the age of 30–35 (or 5–10 years before the earliest known BC in the family). After 75 years, management should be considered on an individual basis. Moreover, for women with a germline *PTEN* PV/LPV, RRM is an option that should be taken into consideration [54].

Recently, germline PV/LPVs in *PIK3CA* and *AKT1* genes have also been reported as being associated with CS [84], but the role of these genes in the pathogenesis of the disease is not well established.

### 2.4. STK11

The *STK11* gene (formerly *LKB1*) is located on chromosome 19p13.3 and encodes a serine/threonine kinase that regulates energy metabolism and cell polarity [85]. The *STK11* gene is a tumor suppressor that is mutated in a variety of sporadic cancers and, for this reason, different therapeutic strategies are under evaluation for the treatment of these tumors [86].

Germline PV/LPVs in the *STK11* gene are associated with the Peutz-Jeghers syndrome [23], an autosomal dominant disorder characterized by melanocytic macules of the lips, buccal mucosa and digits, multiple gastrointestinal hamartomatous polyps, and an increased risk of different tumors. In *STK11* PV/LPV carriers, the lifetime BC risk is estimated to be 32–54%, and the risk of gynecological cancers (cervical, ovarian, uterine) is 13% by the age of 60 [87,88]. The NCCN guidelines suggest that women with *STK11* PV/LPVs should undergo clinical breast examination every 6 months and annual mammography and breast MRI starting at the age of 25 [89]. For the risk of OC, they should undergo pelvic examination and Pap test annually starting at 18–20 years-old [89].

### 2.5. CDH1

The *CDH1* gene is located on chromosome 16q22.1 and encodes the E-cadherin, a transmembrane calcium-dependent protein involved in cell-cell adhesion [90], acting as a tumor suppressor [91] that prevents invasiveness and metastatization [92,93,94].

Germline PV/LPVs in the *CDH1* gene are associated with Hereditary Diffuse Gastric Cancer syndrome (HDGC) [26], an autosomal dominant condition predisposing to diffuse-type gastric cancer (DGC) and lobular breast cancer (LBC). The cumulative risk of LBC for women with a *CDH1* germline PV/LPV is estimated to be 39–52% by 80 years of age [27,95,96,97]. Given the high risk of developing LBC, according to the NCCN guidelines, women with a *CDH1* PV/LPV should undergo annual mammography with consideration of tomosynthesis and breast MRI with contrast starting at the age of 30 [54]. However, there are insufficient data about RRM in these patients; consequently, it is an option that should be evaluated based on family history [54]. At the moment, there are no targeted therapies available for *CDH1* PV/LPV carriers, but drug sensitivities derived from in vitro models of HDGC are under evaluation [98]. Moreover, somatic genetic and epigenetic alterations of the *CDH1* gene occur frequently in sporadic tumors, such as gastric cancer and BC [99], and are associated with poor survival [100]. In particular, recent findings suggest that *CDH1* hypermethylation is a potential novel drug target for developing personalized therapy [101].

## 3. Moderate and Low Penetrance Genes

### 3.1. PALB2, BRIP1, and other Fanconi Anemia Genes

Fanconi anemia (FA) is a genetic disorder, characterized by multiple congenital abnormalities, bone marrow failure, and susceptibility to cancer [34]. The syndrome is caused by biallelic PV/LPVs in one of the 22 FA and FA-like genes that have been identified (except for *FANCB*, which is dominant, being located on the X chromosome) [102]. *BRCA1* and *BRCA2* genes (formerly *FANCS* and *FANCD1*, respectively) are also included in this group, even if the involvement of *BRCA1* in FA has been debated [103] because its biallelic PV/LPVs have been suggested to be lethal during embryonic development [104,105]. However, there are multiple reports of FA patients with *BRCA1* biallelic PV/LPVs, probably due to the retention of a partial activity by the protein [106,107,108,109,110]. Proteins encoded by the FA genes act as tumor suppressors, creating a complex that is activated in case of DNA damage and recruits other proteins involved in DNA repair via HR [111].

Monoallelic PV/LPVs of many FA genes have been reported as being associated with a BC/OC risk, such as *FANCC*, *FANCM*, and some *RAD51* paralogs (see also paragraph 3.5.) [14,112,113,114,115,116,117,118,119,120], but the ones clearly involved in cancer predisposition are *PALB2* and *BRIP1*. Although further studies are required, all these observations suggest that monoallelic PV/LPVs in FA genes are likely to increase the risk of BC/OC [121].

The *PALB2* gene is located on chromosome 16p12.2 and encodes a protein that colocalizes with BRCA2 in nuclear foci, promotes its localization and stability in nuclear structures, and enables its recombinational repair and checkpoint functions [48]. PALB2 binds the single-strand DNA and directly interacts with the recombinase RAD51 to stimulate strand invasion during the HR process [49]. Biallelic PV/LPVs in the *PALB2* gene (formerly *FANCN*) are associated with FA. However, monoallelic PV/LPVs in the *PALB2* gene lead to a higher risk of BC for both sexes [122,123,124,125,126], which has been recently estimated at 53% for females and at 1% for males by the age of 80 [33]. In patients with *PALB2* PV/LPVs, there is also a low increase in the risk of OC [127] and of pancreatic cancer [128,129], which has been estimated at 5% and 2–3%, respectively, by the age of 80 [33]. The NCCN guidelines suggest that women with *PALB2* PV/LPVs should undergo annual mammography with consideration of tomosynthesis and breast MRI starting at the age of 30 [54]. Moreover, RRM is an option that should be considered, whereas data about RRSO are insufficient, and family history should be taken into account [54].

The *BRIP1* gene is located on chromosome 17q23.2 and encodes a DNA helicase that binds directly to the BRCT repeats of BRCA1 and is necessary for efficient DSB repair in the HR process [130,131]. Biallelic PV/LPVs in the *BRIP1* gene (formerly *FANCJ*) are also associated with FA. However, monoallelic PV/LPVs in the *BRIP1* gene lead to a higher risk of OC that is estimated to be around 6% during a lifetime [127,132]. In contrast to the *PALB2* gene, the risk of BC for *BRIP1* PV/LPV carriers is estimated to be low or none [133,134,135], and there is insufficient evidence for risk management [54]. For the risk of OC, according to the NCCN guidelines, RRSO is an option that should be evaluated at 45-50 years or earlier based on family history [54].

Given the involvement of the FA complex in the HR process, some clinical trials are evaluating the efficacy of PARP inhibitors in patients with metastatic BC and germline alterations of *PALB2*, *BRIP1*, and other FA genes such as *FANCA*, *FANCC*, *FANCD2*, *FANCE*, *FANCF*, and *FANCM* [136]. Moreover, germline and somatic alterations in genes involved in HR can predict platinum response and survival in ovarian, fallopian tube, and peritoneal carcinomas [137], giving new therapeutic opportunities for these tumors.

### 3.2. ATM

The *ATM* gene is located on chromosome 11q22.3 and encodes a phosphatidylinositol 3-kinase protein that acts as a tumor suppressor and responds to DNA damage by phosphorylating key substrates involved in DNA repair and cell cycle control [138].

Biallelic PV/LPVs in the *ATM* gene are associated with ataxia-telangiectasia [28], an autosomal recessive disorder characterized by cerebellar ataxia, telangiectases, immune defects, and a predisposition to malignancy [28,139]. However, monoallelic PV/LPVs are associated with an increased risk of BC that is estimated to be 17–52% during a lifetime [140,141,142,143,144,145]. The NCCN guidelines suggest that women with an *ATM* PV/LPV should undergo annual mammography with consideration of tomosynthesis and breast MRI with contrast starting at the age of 40 [54]. There is insufficient evidence about RRM in these patients and this option should be evaluated based on family history [54]. Moreover, given the involvement of ATM in the DNA repair processes, there are clinical trials that are evaluating the efficacy of PARP inhibitors in patients with *ATM* germline variants and metastatic BC [136]. In addition to germline variants, somatic *ATM* alterations commonly occur in a number of sporadic human cancers, in particular leukemias and carcinomas of the breast and lung [146], and these findings generate new opportunities for the future treatment of these tumors.

### 3.3. CHEK2

The *CHEK2* gene is located on chromosome 22q12.1 and encodes a nuclear Ser/Thr kinase that acts as a tumor suppressor in different cellular processes [147]. In response to DSBs, CHEK2 protein is phosphorylated by ATM and catalyzes the phosphorylation of CDC25C, down-regulating it and preventing entry into mitosis [148]. Furthermore, after DNA damage, CHEK2 phosphorylates the p53 tumor suppressor protein and prevents its degradation, leading to cell cycle arrest in G1 [149]. Under gamma irradiation, CHEK2 also phosphorylates BRCA1 on Ser-988, activating the DNA repair process [150]. Finally, CHEK2 has been shown to induce apoptosis independently from p53, via phosphorylation of the PML (promyelocytic leukemia protein) tumor suppressor [151].

The first *CHEK2* germline PV/LPVs identified have been associated with the Li-Fraumeni syndrome [152,153]; subsequently, this association has been questioned because of phenotype differences among LFS patients and *CHEK2* PV/LPV carriers [154]. Germline PV/LPVs in this gene are associated with an increased risk of BC [155,156,157,158], which is estimated to be 25–39% during a lifetime [29,159,160]. In particular, *CHEK2* variant c.1100delC is associated with a two- to three-fold increase in BC risk in women and a ten-fold increase of risk in men [161,162,163]. In accordance with the NCCN guidelines, women with a *CHEK2* PV/LPV should undergo annual mammography with consideration of tomosynthesis and breast MRI with contrast starting at the age of 40 [54]. However, there is insufficient evidence about RRM in these patients, and family history should be considered [66]. In addition to BC, *CHEK2* PV/LPVs have also been associated with other cancers [164], including prostate [165,166,167], colorectal [168], and gastric cancers [169]. PV/LPVs in the *CHEK2* gene have also been linked to OC risk [17,170], but this association has been debated for a long time [73,171,172,173,174], and there are insufficient data to recommend a surveillance protocol or RRSO [54]. Moreover, given the involvement of CHEK2 in DNA repair processes, there are clinical trials that are evaluating the efficacy of PARP inhibitors in patients with *CHEK2* germline variants and metastatic BC [136]. In addition to germline variants, somatic *CHEK2* alterations commonly occur in sporadic cancers [175], and these findings pave the way for the development of new targeted therapies.

### 3.4. BARD1

The *BARD1* gene is located on chromosome 2q35 and encodes a protein that interacts with the N-terminal region of BRCA1 [176] and acts as a tumor suppressor creating a BRCA1/BARD1 heterodimer with ubiquitin E3 ligase activity [177].

PV/LPVs in the *BARD1* gene have been associated with an approximately two-fold increase of lifetime BC risk [12,178,179,180,181,182], but the penetrance has not been estimated precisely and a surveillance protocol is not routinely recommended [54]. Moreover, *BARD1* PV/LPVs have also been linked to OC risk [17,179], but this association has been recently excluded [30,127]. Given the involvement of BARD1 in the HR process and its association with the BRCA1 protein, there are clinical trials that are evaluating the efficacy of PARP inhibitors in patients with germline *BARD1* variants and metastatic BC [136]. Moreover, somatic alterations of the *BARD1* gene have been identified in different tumors, such as BC and uterine cancer [183], generating new opportunities for the future treatment of these tumors.

### 3.5. RAD51C, RAD51D, and other RAD51 Paralogs

The *RAD51* gene encodes a protein involved in the repair of DSBs during the HR process. In mammals, seven *RAD51* paralogs have been identified: *RAD51*, *RAD51B*, *RAD51C*, *RAD51D*, *XRCC2*, *XRCC3,* and *DMC1* [184]. The paralogs form two identified complexes: BCDX2 (RAD51B-RAD51C-RAD51D-XRCC2) and CX3 (RAD51C-XRCC3), which act in different stages of HR: The BCDX2 complex is responsible for the recruitment or stabilization of RAD51 at damage sites and the CX3 complex acts downstream of this process [185].

Biallelic PV/LPVs of the *RAD51*, *RAD51C*, and *XRCC2* genes (formerly *FANCR*, *FANCO*, and *FANCU*, respectively) are associated with FA (see paragraph 3.1.) [34]. In contrast to this, monoallelic variants of the *RAD51* gene and of five of its paralogs have recently been involved in cancer predisposition: *RAD51B*, *RAD51C*, and *RAD51D* in OC; *RAD51*, *RAD51B*, and *XRCC2* in BC [35,186,187,188,189,190,191,192,193,194]. However, the two genes well known to be associated with cancer risk are *RAD51C* and *RAD51D* [36,37,195].

The *RAD51C* gene is located on chromosome 17q22 and encodes a protein that acts in HR creating complexes with other RAD51 paralogs [185]. The lifetime risk of OC for *RAD51C* PV/LPV carriers is estimated to be around 7%, whereas the BC risk is controversial [35,36].

The *RAD51D* gene is located on chromosome 17q12 and encodes a protein that, as well as RAD51C, interacts with other RAD51 paralogs in the process of HR [196]. The lifetime risk of OC for *RAD51D* PV/LPV carriers is estimated to be around 15%, whereas the BC risk is controversial [35,37].

The lifetime risk of OC in carriers of PV/LPVs of the *RAD51C* and *RAD51D* genes appears to be sufficient to justify consideration of RRSO, which, according to NCCN guidelines, should be held around the age of 45–50, or earlier based on family history [54]. Germline and somatic alterations in HR genes predict platinum response and survival in ovarian, fallopian tube, and peritoneal carcinomas [137], and there are clinical trials that are evaluating the efficacy of PARP inhibitors in patients with germline *RAD51C/D* variants and metastatic BC [136].

### 3.6. MRN Complex

The MRN complex (MRE11-RAD50-NBS1) is a complex of three proteins (encoded by *MRE11*, *RAD50*, and *NBN* genes) involved in the repair of DSBs and in telomere maintenance [197]. These proteins are known tumor suppressors because loss of function of any of them results in genome instability, and defective MRN function has been linked to many types of cancer, including breast, ovarian, colorectal, gastric, and prostate cancers, as well as leukemia and melanoma [197]. Germline variants in these three genes have also been associated with an increased risk of BC/OC [137,198,199,200,201]; in particular, the *NBN* gene, located on chromosome 8q21.3, which encodes the Nibrin protein, also known as p95 and NBS1 [202], which is responsible for the localization of the complex and for the interactions with other DSB-signaling and DNA-repair proteins [197].

Biallelic PV/LPVs of *NBN* are the cause of Nijmegen breakage syndrome (NBS), an autosomal recessive disease characterized by chromosomal instability that determines progressive microcephaly, intrauterine growth retardation and short stature, recurrent sinopulmonary infections, an increased risk for cancer, and premature ovarian failure in females [32]. *RAD50* and *MRE11* biallelic PV/LPVs have also been associated with an NBS-like disorder [203,204].

On the other hand, *NBN* monoallelic PV/LPVs predispose to the development of BC, prostate cancer, medulloblastoma, and melanoma [205,206,207,208,209,210,211]. In particular, women with the 657del5 variant have a higher risk of BC, and NCCN guidelines suggest that they should undergo annual mammography with consideration of tomosynthesis and breast MRI with contrast starting at the age of 40. Of note, current data suggest that BC risk is not increased for carriers of PV/LPVs other than 657del5 [54]. *NBN* germline PV/LPVs have also been identified in patients with OC, but the link between *NBN* alterations and OC risk is still under evaluation [17,127,137,212], and there are insufficient data to recommend a surveillance protocol or RRSO [54]. Moreover, given the involvement of the MRN complex in the HR, there are clinical trials that are evaluating the efficacy of PARP inhibitors in patients with *NBN*, *MRE11*, and *RAD50* variants and metastatic BC [136].

Variants in *RINT1* gene have also been associated with BC risk [213]. This gene encodes a protein that was first identified for its ability to interact with the RAD50 protein in the regulation of cell cycle progression and telomere length [214]. This association is intriguing given the RINT1 protein function, but it needs to be investigated further.

### 3.7. Mismatch Repair Genes

Lynch syndrome (LS) is an autosomal dominant disorder, characterized by a high risk of developing colorectal and endometrial cancers [38]. LS is caused by PVs in the *MLH1*, *MSH2*, *MSH6*, and *PMS2* genes, coding the proteins of the DNA mismatch repair (MMR) [215], or by large deletions of the *EPCAM* gene, located upstream of the *MSH2* gene [216]. MMR proteins work coordinately in sequential steps to initiate repair of DNA mismatches, constituted by erroneous insertions, deletions, and substitutions of bases that can arise during DNA replication and recombination [217].

Patients with LS also have an increased risk of developing other tumors, in particular, they have a lifetime risk of OC that is estimated to be 4–12% [218,219]. Unlike OCs associated with PV/LPVs in *BRCA1/2* genes, which are mainly of the serous type, those associated with LS are more likely to be endometrioid or clear cell [220]. Whether LS is directly related to BC predisposition is currently a matter of debate [115,218,221,222,223,224]: PVs of the MMR genes have been reported in patients with BC [117,225], but a consistent association between LS and BC has never been demonstrated [226,227,228].

Regarding surveillance for LS patients, the data do not support routine OC screening (transvaginal ultrasound and serum CA-125 testing may be considered but have not been shown to be sufficiently sensitive or specific to warrant a routine recommendation). In accordance with the NCCN guidelines, bilateral RRSO is an option that may be considered and individualized based on whether childbearing is complete, menopausal status, comorbidities, family history, and LS gene, as risks for OC vary by the mutated gene (there is insufficient evidence to recommend RRSO in *MSH6* and *PMS2* PV/LPV carriers) [89]. Given the uncertain risk of BC in LS patients, breast surveillance is not recommended but should be evaluated based on family history [89].

Moreover, patients with LS, who have a deficiency of the MMR system (dMMR), can benefit from chemoprevention based on the use of daily aspirin [229] and can be treated with anti-PD-1/PD-L1 therapy [230,231]. The dMMR also arises in sporadic cancers through genetic or epigenetic mechanisms [232] and is observed in a reasonable proportion of OCs [233], generating important implications on the treatment and prognosis [234].

### 3.8. NF1

The *NF1* gene is located on chromosome 17q11.2 and encodes Neurofibromin, a cytoplasmic protein that regulates several intracellular processes, including the Ras-cAMP (cyclic adenosine monophosphate) pathway, the ERK (extracellular signal-regulated kinase)/MAPK cascade, adenylyl cyclase, and cytoskeletal assembly [235]. In particular, Neurofibromin has been shown to increase the rate of guanosine triphosphate (GTP) hydrolysis of Ras and act as a tumor suppressor by reducing Ras activity [236,237].

Germline PV/LPVs in the *NF1* gene are responsible for Neurofibromatosis type I, an autosomal dominant syndrome characterized by multiple *café au lait* spots, axillary and inguinal freckling, multiple cutaneous neurofibromas, iris Lisch nodules, and choroidal freckling [31].

Women with *NF1* PV/LPVs have a substantially increased risk of developing BC before the age of 50 [14,238,239,240,241] and of dying of BC [242]. In these patients, according to the NCCN guidelines, mammography with consideration of tomosynthesis should be performed annually beginning at the age of 30, and annual breast MRI with contrast should be considered between the ages of 30 and 50 [54]. However, the efficacy and cost-effectiveness of such surveillance have not yet been demonstrated [243].

In addition to germline variants, somatic *NF1* alterations commonly occur in sporadic cancers and are associated with increased cancer risk and drug resistance [244]. This generates implications even on the treatment of these tumors because, in NF1-deficient tumors, some drugs, such as MEK inhibitors, have shown their efficacy [245].

## 4. Other Emerging Genes

Thanks to the wide use of NGS, the number of genes that have been associated with a BC/OC risk has hugely increased in the last few years. However, the most critical issue is the penetrance estimate of the penetrance of these PV/LPVs.

Recently, PV/LPVs in genes coding RecQ helicases have been reported in patients with BC/OC [117,246,247,248,249,250,251,252,253,254]. Interestingly, PV/LPVs in three of these genes are responsible for autosomal recessive genetic syndromes, all associated with an increased risk of malignancies: In fact, *BLM*, *WRN*, and *RECL4* genes are the genetic cause of Bloom syndrome [255], Werner syndrome [256], and Rothmund–Thomson syndrome [257], respectively.

The proteins encoded by the *ABRAXAS1* and *UIMC1* genes (also known as *FAM175A* and *RAP80*) form a complex with BRCA1, required for DNA damage response [258]. PV/LPVs in both genes have been linked to an increased risk of BC, but penetrance estimates are still missing [259,260].

Other genes, such as *PPM1D*, *ERCC3*, *BAP1*, and *NTHL1* have been reported in BC/OC families [117,261,262,263,264,265,266], but further studies are needed to confirm these associations.

Moreover, in 2018, a systematic review of The Cancer Genome Atlas (TCGA) data was performed, focusing on pathogenic germline variants: besides the *BRCA1* and *BRCA2* genes, PV/LPVs in *ATM*, *PALB2*, *RET*, *NF1*, *TP53*, *BUB1B*, *MAX*, and *APC* genes have been detected in BC patients, and PV/LPVs in *ATM*, *PALB2*, *TP53*, *NF1*, and *SDHA* genes have been detected in OC patients [267]. Some of these genes are intriguing candidates in the predisposition to BC/OC and, in the future, could be included in diagnostic panels for the assessment of cancer risk.

## 5. Conclusions

BC and OC are both in the top 10 of the most common and deadly tumors for women and, among risk factors for the development of these cancers, genetic predisposition plays an important role.

*BRCA1* and *BRCA2* have been known for decades to be predisposition genes to BC and OC. Consequently, for PV/LPV carriers in these genes, accurate cancer risk estimates are available, as well as surveillance protocols for cancer prevention and early detection of the disease.

New genes are constantly emerging from NGS studies [10,14,16,117,180,268,269,270,271,272], showing that BC/OC predisposition is distributed over many genes, with only a few genes being recurrently mutated. However, testing on genes other than *BRCA1* and *BRCA2* is not routinely performed, due to lack of information about the actual risks for PV/LPV carriers and the unavailability of surveillance programs.

Besides *BRCA1* and *BRCA2*, there are some genes with high penetrance, such as *TP53*, *PTEN*, *STK11*, and *CDH1*, whose variants are associated with other cancer genetic syndromes (Table 1) that, however, also include a high risk of BC and/or OC. Consequently, these genes need to be included in a panel of genes for the identification of patients at risk of BC/OC. Moreover, there are some other genes, such as *PALB2*, *BRIP1*, *ATM*, *CHEK2*, *BARD1*, *RAD51C*, *RAD51D*, *NBN*, *NF1*, and MMR genes that are classified as moderate or low penetrance genes for the risk of BC/OC (Table 1), but the increasing evidence of associated cancer risks and the availability of recommendations for the management of the variant carriers suggest that they should be included, whenever possible, in a gene panel for BC/OC predisposition.

In particular, *PALB2* is emerging as the most important BC predisposition gene after *BRCA1* and *BRCA2* [122,123], but at the same time, it is still classified as a moderate penetrance gene. In the last few years, many studies pinpointed this gene as being associated with a high risk of BC and a high rate of bilateral BC [117]. These observations further highlight both the high risk of BC associated with *PALB2* PVs and the importance of adding the *PALB2* gene to standard genetic tests for patients with suspected HBOC syndrome.

NGS-based approaches have also highlighted unexpected overlapping among genetic syndromes predisposing to BC/OC and gastrointestinal tumors, such as colorectal, gastric, and pancreatic cancers, raising the question of phenotypes associated with individual cancer susceptibility genes [273,274]. These findings address the choice of wide panels, including the genes involved in the main cancer syndromes that can be used independently of the personal and family history of cancer. This creates new diagnostic opportunities but also increases the risk of an incorrect genetic diagnosis [275].

On the whole, it is indisputable that NGS has deeply increased our knowledge of BC predisposition by increasing the number of susceptibility genes. However, because of the growing demand for higher throughput and lower costs, quality data and standardized procedures need to be carefully assessed. Moreover, genetic counseling and risk evaluation, as well as clinical management of patients and families at risk, are becoming more and more challenging [276]. In particular, all health-care professionals who offer genetic testing must engage in constant education as the field is continuously evolving, with new data becoming available [277]. An important aspect is the selection of patients for the genetic test, which is currently based on the number and type of cancers in the family and on the age of onset of these tumors. Several models are available to estimate the probability of having a *BRCA1/2* variant [278,279,280,281,282,283,284]. These models can be used to select patients for a multigene panel test for the risk of BC/OC but, with the increase in the number of predisposing genes, they should be improved to identify individuals who can really benefit from a genetic test and, at the same time, to avoid the overuse of genetic tests.

Moreover, another important issue for patients with a cancer predisposition syndrome is prophylactic surgery. To date, we know that prophylactic surgery is an option that should be evaluated by patients with specific syndromes. In women with *BRCA1/2* PV/LPVs, bilateral RRM and bilateral RRSO have been demonstrated as effective measures to prevent cancer [285]. In individuals with LS, prophylactic hysterectomy and bilateral RRSO can be considered after childbearing is completed, whereas prophylactic colectomy before the development of colon cancer is generally not recommended because screening colonoscopy with polypectomy is an effective preventive measure [286]. Finally, for individuals with *CDH1* PV/LPVs, prophylactic gastrectomy should be strongly advised; however, there are insufficient data about prophylactic mastectomy [287]. For individuals with PV/LPVs in other cancer genes, more data are needed to perform a risk-benefit assessment of prophylactic surgeries but, for some genes, guidelines suggest that RRM and/or RRSO should be evaluated in accordance with family history and, in particular, the age of onset of the tumors in the family [54,89].

Recently, cancer genetic predisposition and precision medicine have found a contact point, thanks to the discovery of the therapeutic potential of PARP inhibitors in *BRCA1/2* PV/LPV carriers [288,289], at first in OC [290] and then in BC, prostate, and pancreatic cancers [291,292,293]. PARP inhibitors have shown their efficacy, not only in patients with germline and somatic *BRCA1/2* alterations [57], but also in patients with PV/LPVs in genes involved in the HR pathway with BRCA1 and BRCA2 proteins [294,295,296,297], and clinical trials are currently addressing this [136]. These results pave the way for the future use of PARP inhibitors in all tumors with a deficiency of the HR system, independently of the germline or somatic nature of the alteration.

These “personalized” treatments are possible thanks to the fact that tumors originating in individuals with a germline PV/LPV in a cancer predisposition gene usually have a specific “mutational signature” that reflects the pathway in which the gene is involved [298]. Indeed, the main genes associated with BC/OC predisposition encode proteins that act as tumor suppressors being involved mostly in DNA damage repair processes, such as HR and MMR, and are strictly linked together. This aspect will become very important in the future because it can help in the identification of new genes involved in the predisposition and can guide the choice of the best therapeutic approach in terms of targeted therapies, chemoprevention, and prophylactic surgeries for a medicine personalized on the genetic characteristics of the patient.

The diagnostic use of multigene panels, instead of the traditional single-gene analysis, generates many advantages as well as some critical issues. Before the advent of NGS, turn-around times for genetic testing were long, in some cases more than 6–12 months, while nowadays new technologies provide results in less than a month in many cases. This short time is extremely useful for the affected individuals because the result of the genetic test can address or modify the surgical and therapeutic approach to the disease but, at the same time, it can generate issues in the genetic counseling and the management of the family, since the implications of cancer predisposition need time to be understood properly by the patient and by family members. The use of large gene panels can also create an increase in the number of false-positive findings [299]. For this reason, a second confirmatory analysis is always recommended, possibly with another technique, such as Sanger sequencing [300]. Another important issue is the interpretation of variants of uncertain significance (VUS), whose number increases exponentially with the increase of tested genes. Of note, the assessment of the pathogenicity of the genetic variants is based on guidelines that take into account many factors [301], but this classification refers to the potential role in cancer development without taking into account the penetrance and the spectrum of the associated diseases. Many non-easily classifiable variants are identified by NGS studies and, although several techniques can now be used to investigate their pathogenicity [302], efficient and accurate classification methods are still needed to translate theoretical information into clinical practice. The bioinformatics tools for the prediction of pathogenicity seem inadequate to classify many variants and to identify higher-risk patients [117]. However, risk assessment of candidate variants is made difficult by the limited number of variant carriers and by the possible interference of different genetic and environmental factors. The multifactorial nature of BC/OC and the presence of predisposing variants in genes never included in panels for standard genetic tests are likely to further increase the complexity of the scenario.

Taking into account all these considerations, it is clear how difficult it is to find the right combination of genes to be tested in patients with a suspected genetic predisposition to BC/OC. At the international level, efforts are being made to achieve a consensus [303,304,305,306], but the identification of a balance between costs for health systems and benefits for patients remains one of the biggest challenges for the future. Moreover, larger case-control studies are needed to better refine the penetrance estimates and to evaluate the correct preventive and therapeutic approach for each patient.

## Figures and Tables

**Table 1:** List of the main genes associated with breast cancer (BC)/ovarian cancer (OC) with associated syndromes and BC/OC risk estimates.

Syndrome Associated	Gene	Locus	BC Risk *	OC Risk *	References
Hereditary Breast and Ovarian Cancer (AD)	*BRCA1*	17q21.31	57–65% (by age 70)	39–44% (by age 70)	[20,21,22]
	*BRCA2*	13q13.1	45–55% (by age 70)	11–18% (by age 70)	
Peutz-Jeghers syndrome (AD)	*STK11*	19p13.3	32–54%	18–21%	[23]
Cowden syndrome (AD)	*PTEN*	10q23.31	25–85%	NA	[24]
Li-Fraumeni syndrome (AD)	*TP53*	17p13.1	25–79%	NA	[25]
Hereditary Diffuse Gastric Cancer (AD)	*CDH1*	16q22.1	39–52% (by age 80)	NA	[26,27]
Ataxia–telangiectasia (AR)	*ATM*	11q22.3	17–52%	NA	[28]
-	*CHEK2*	22q12.1	25–39%	NA	[29]
-	*BARD1*	2q35	NA	NA	[30]
Neurofibromatosis type I (AD)	*NF1*	17q11.2	~8% (by age 50)	NA	[31]
Nijmegen breakage syndrome (AR)	*NBN*	8q21.3	12–30%	NA	[32]
Fanconi anemia (AR)	*PALB2*	16p12.2	44–63% (by age 80)	2–10% (by age 80)	[33]
	*BRIP1*	17q23.2	NA	~6%	[34]
-	*RAD51C*	17q22	NA	~7%	[35,36]
-	*RAD51D*	17q12	NA	~15%	[35,37]
Lynch syndrome (AD)	*MLH1*	3p22.2	NA	4–12%	[38]
	*MSH2*	2p21-p16			
	*MSH6*	2p16.3			
	*PMS2*	7p22.1			
	*EPCAM*	2p21			

AD: autosomal dominant; AR: autosomal recessive; NA: not assessed. * The percentages represent lifetime risks unless otherwise specified.

## Abbreviations

AD	Autosomal dominant
AR	Autosomal recessive
BASC	BRCA1-associated genome surveillance complex
BC	Breast cancer
cAMP	Cyclic adenosine monophosphate
DGC	Diffuse-type gastric cancer
DSB	Double-strand break
ERK	Extracellular signal-regulated kinase
FA	Fanconi anemia
GTP	Guanosine triphosphate
HBOC	Hereditary Breast and Ovarian Cancer
HDGC	Hereditary Diffuse Gastric Cancer syndrome
HR	Homologous recombination
LBC	Lobular breast cancer
LFS	Li-Fraumeni syndrome
LPV	Likely-pathogenic variant
LS	Lynch syndrome
MAPK	Mitogen-activated protein kinase
MBC	Male breast cancer
MMR	Mismatch repair
MRI	Magnetic resonance imaging
MRN	MRE11-RAD50-NBS1 complex
NA	Not assessed
NBS	Nijmegen breakage syndrome
NCCN	National Comprehensive Cancer Network
NGS	Next-Generation Sequencing
OC	Ovarian cancer
PARP	Poly adenosine-diphosphate ribose polymerase
PJS	Peutz-Jeghers syndrome
PML	Promyelocytic leukemia protein
PV	Pathogenic variant
RR	Relative risk
RRM	Risk-reducing mastectomy
RRSO	Risk-reducing salpingo-oophorectomy
TCGA	The Cancer Genome Atlas
VUS	Variant of uncertain significance
